# Gouy phase effects on photocurrents in plasmonic nanogaps driven by single-cycle pulses

**DOI:** 10.1515/nanoph-2023-0897

**Published:** 2024-04-15

**Authors:** Andrea Rossetti, Matthias Falk, Alfred Leitenstorfer, Daniele Brida, Markus Ludwig

**Affiliations:** Department of Physics and Materials Science, 81872 University of Luxembourg, Luxembourg, Luxembourg; Department of Physics and Center for Applied Photonics, 26567 University of Konstanz, Konstanz, Germany; 28332 Deutsches Elektronen-Synchrotron (DESY), Hamburg, Germany

**Keywords:** Gouy phase, plasmonic nanogap, field-emission, strong-field physics, carrier-envelope phase

## Abstract

The investigation of optical phenomena in the strong-field regime requires few-cycle laser pulses at field strengths exceeding gigavolts per meter (GV/m). Surprisingly, such conditions can be reached by tightly focusing pJ-level pulses with nearly octave spanning optical bandwidth onto plasmonic nanostructures, exploiting the field-enhancement effect. In this situation, the Gouy phase of the focused beam can deviate significantly from the monochromatic scenario. Here, we study the effect of the Gouy phase of a pulse exploited to drive coherent strong-field photocurrents within a plasmonic gap nanoantenna. While the influence of the specific Gouy phase profile in the experiment approaches the monochromatic case closely, this scheme may be utilized to identify more intricate phase profiles at sub-diffraction scale. Our results pave the way for Gouy phase engineering at picojoule (pJ) pulse energy levels, enabling the optimization of strong-field optical phenomena.

## 1 Introduction

The developments in the generation of few-cycle laser pulses have made it possible to expand the experimental investigation of light–matter interaction into the strong-field regime [1], [2], [3], [4], [5]. This is roughly attained when the amplitude of the laser electric field reaches values comparable to the conditions seen by the electrons in a crystal lattice or molecule. Under such conditions, light–matter interaction cannot be modeled within the traditional perturbative approach, where the response of the material to the applied field shows a polynomial dependence on the laser intensity. Instead, in the strong-field regime, it is the exact profile of the electric field transient that governs the interaction process. In particular, the effect of the carrier-envelope phase (CEP) in phenomena such as high-harmonic generation or field-emission from metal surfaces has been extensively studied [6], [7], [8], [9]. In practice, electric field strengths of roughly 10 V/nm are needed to access the strong-field regime [10]. Those conditions may be reached in free space with multi-mJ few-cycle pulses under focusing. Significantly relaxed conditions are required when leveraging the plasmonic field enhancement of sharp metal tips or resonant nanostructures, where even pJ-level pulses are sufficient to drive strong-field photoionization [8], [10], [11], [12], [13]. In this context, we investigate the role of the Gouy phase shift in the highly nonlinear process occurring when a tightly focused few-cycle laser pulse drives current transport inside a planar plasmonic nanostructure with a few-nm gap.

It is well known [14] that a focused monochromatic electromagnetic wave experiences a phase shift along the propagation direction through the beam waist, called Gouy phase shift, equal to $\Delta \phi \left(z\right)=-\text{atan}\left(\frac{z}{z_{R}}\right)$ , where *z* is the propagation distance and *z*_*R*_ is the Rayleigh range. From a conceptual viewpoint, the application of this model to CEP-dependent nonlinear interactions is delicate. In fact, the Gouy model is only exact for monochromatic waves, while the few-cycle pulses typically employed for strong-field experiments feature bandwidths spanning roughly one octave. Since the Rayleigh range usually depends on the wavelength, the different spectral components of a focused laser pulse will individually experience different phase shifts during propagation. Because of this, for few-cycle laser pulses, significant deviations can be expected with respect to the case of a monochromatic Gaussian beam [15], [16].

Using a diffraction-based approach, a generalized analytical model has been proposed by Porras to describe the spatially dependent phase shift $\Delta \phi \left(z,r\right)$ of a fundamental but broadband Gaussian mode [17], [18]:

(1) $$\Delta \phi \left(z,r\right)=-\text{atan}\left(\frac{z}{z_{R}}\right)+g\frac{1-2{\left(\frac{r}{w\left(z\right)}\right)}^{2}}{\frac{z}{z_{R}}+\frac{z_{R}}{z}}$$

where *z*_*R*_ is the Rayleigh range at the central wavelength, $w\left(z\right)$ is the beam size, *r* is the radial coordinate, and *g* is a dimensionless factor dependent on the properties of the input beam (that is the beam prior to focusing) and defined as

(2) $$g={\left.\frac{d\zeta_{R}}{d\omega }\right|}_{\omega_{0}}\;\frac{\omega_{0}}{\zeta_{R}\left(\omega_{0}\right)}$$

Here, $\zeta_{R}\left(\omega \right)$ is the frequency-dependent Rayleigh range of the beam prior to focusing and *ω*_0_ is the central angular frequency of the laser. Like in a monochromatic Gaussian beam, the electromagnetic field undergoes a total phase shift Δ*ϕ* = π while passing through the focal plane. However, the spatial features of the phase shift strongly depend on the frequency-dependent geometrical properties of the input beam. If the various spectral components all propagate with the same Rayleigh range, the behavior of the CEP upon focusing can still be modeled within the monochromatic Gouy picture. Instead, when the Rayleigh range of the incoming laser beam is dispersive, the correction term in equation (1) cannot be neglected. The deviation from the monochromatic Gouy phase is quantified by the dimensionless parameter *g*, which is proportional to the frequency derivative of the Rayleigh range *ζ*_*R*_ of the input beam, evaluated at the central frequency of the spectrum. To a first degree of approximation, the effect of the g parameter is to renormalize the Rayleigh range *z*_*R*_ associated to the phase shift. Around the focal plane, equation (1) can be well approximated by a monochromatic Gouy shift with effective Rayleigh range $z_{R}^{\prime }=\frac{z_{R}}{1-g}$ . As a benchmark, $\left|g\right|=0.1$ corresponds to a 10 % variation of the effective Rayleigh with respect to the monochromatic value. Assessing the spatial dependence of the CEP becomes particularly relevant whenever the relative dimensions of beam and sample are such that the phase of the electric field cannot be considered uniform over the interaction area. Some experiments [16], [19], [20], [21], [22] have already investigated the CEP evolution of focused broadband pulses, showing nontrivial spatial profiles. In particular, it has recently been demonstrated that different parameters like chromatic aberrations and chirp can strongly affect the spatial variations of the CEP in the vicinity of the focus [22], [23], [24].

Plasmonic nanostructures have proven to be a successful platform to study strong-field phenomena. Other than enhancing the field in the vicinity of the nanostructure, the plasmonic resonance also causes spectral and temporal distortions with respect to the incoming laser transients [25]. In this context, we propose to investigate how the Gouy CEP shift of focused octave-spanning single-cycle laser pulses affects the field-emission inside a plasmonic nanogap.

## 2 Methods

**Figure 1:**
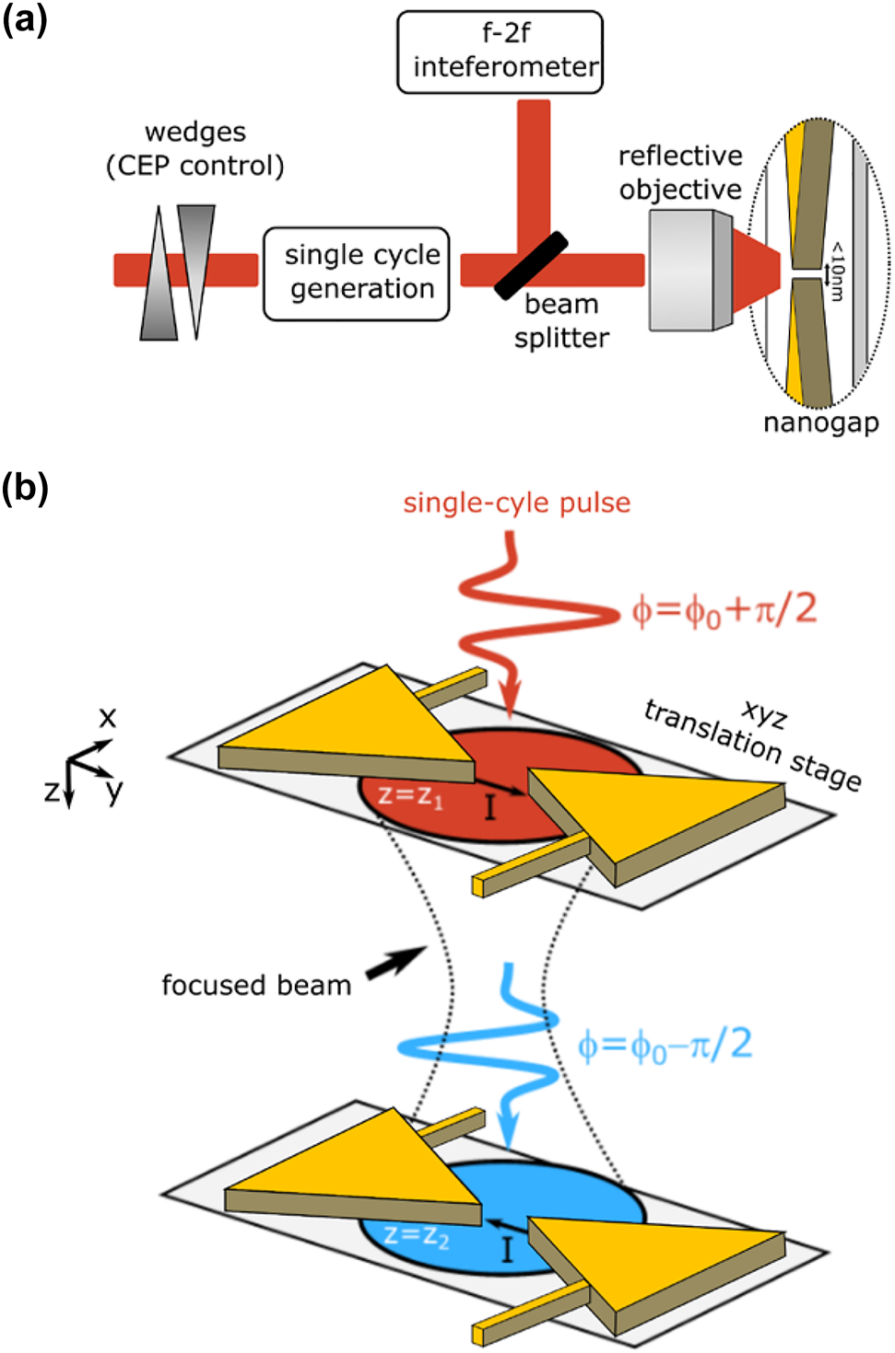
Schematic representation of the experiment: (a) passively CEP-stabilized laser pulses from an Er:fiber laser system [23] are spectrally broadened and compressed to achieve single-cycle laser pulses. Part of the total power goes to an f-2f interferometer. The f-2f interferometer, in combination with a pair of fused silica wedges placed in the beam path before spectral broadening, allows control of the CEP without distortion of the pulse envelope. The transmitted beam is coupled onto the nanogap of a bowtie antenna by means of a 0.52NA reflective objective. (b) The bowtie antenna is mounted on an *xyz* translation stage. By moving the antenna with respect to the beam focus, we can investigate how the spatial profile of the CEP affects the field-emission process.

When single-cycle laser pulses are coupled into the junction of a few-nanometer gap Au bowtie antenna, field-induced electron currents are generated across the nano-junction [26]. Because of the high plasmonic field enhancement taking place at the junction, only a few picojoules of pulse energy are needed to generate a detectable current [13]. In this process, the laser electric field transient acts as an optical bias, causing nonperturbative field emission and subsequent ballistic acceleration of electrons across the nanogap. The amplitude and direction of the generated current is controlled by the CEP of the driving laser field. When the field is cosine shaped, a net electron current can be measured due to the highly nonlinear response of the junction, while no net-current is generated in the case of an antisymmetric sine-like transient. More generally, a full oscillation of the field-induced current can be observed by sweeping the CEP over 2π, with total inversion of the maximum that is obtained when a cosine pulse changes polarity (see Figure 1(b)). In this work, we employ CEP-stable single-cycle laser pulses from a home-made Er:fiber laser system operating at a repetition rate of 40 MHz [27] to drive field-emission from individual Au bowtie nanoantennas. The generation of the pulses comprises different steps. First of all, laser pulses from an Er:fiber oscillator are amplified and passively CEP stabilized. The CEP-stable pulses are then again amplified and spectrally broadened in a highly nonlinear fiber, to achieve an octave-spanning bandwidth centered around 1.57 μm. The CEP can be controlled by varying the insertion of a fused-silica wedge pair and is monitored with an f-2f interferometer [28]. Since the CEP control occurs before the reamplification stage and the final spectral broadening, no temporal distortion of the laser pulses takes place. After compression, the pulses are characterized with two-dimensional shearing interferometry [29]. The measured time duration of 5 fs corresponds to a single oscillation period of the carrier-wave electric field. A schematic representation of the experiment is shown in Figure 1. The single-cycle laser transients with 10-pJ pulse energy are coupled onto the nanoantenna junction by means of a 0.52NA reflective objective, thus avoiding temporal distortion of their waveform. The placing of the nanoantenna can be controlled with a precision of 50 nm by means of a three-axis closed loop nano-positioner. The position of the focal plane is determined by means of the knife-edge method, using a lithographically defined metallic wire in the vicinity of the antenna. Fine optimization of the nanoantenna position with respect to the focal plane is then achieved by maximizing the amplitude of the CEP-dependent current.

**Figure 2:**
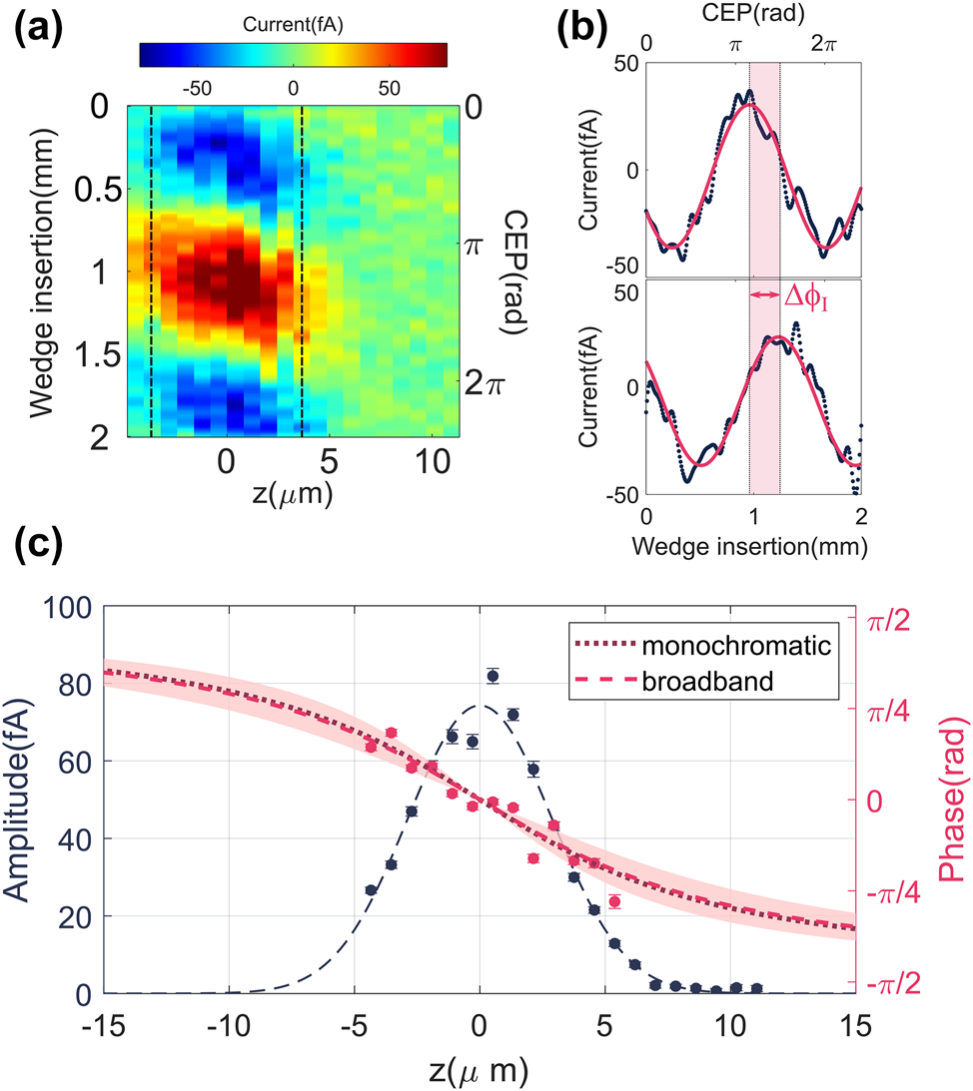
Measured evolution of *ϕ*_*I*_ along the focal axis. (a) Map showing the field-emitted current as a function of wedge insertion and position along the focal axis. For each *z* position, the wedge insertion is varied so as to scan the CEP of the incoming laser pulse over more than 2π. (b) Vertical cuts (blue dots) of the 2d map at the two positions indicated by the dashed lines. Fit (pink solid curve) of the experimental data, from which amplitude and phase of the current oscillation are extracted. The pink shaded region highlights the phase shift Δ*ϕ*_*I*_ detected in the current oscillation. (c) Amplitude (blue circles) and phase (pink circles) of the current oscillation as a function of position along the focal axis. The amplitude data points are fitted with a Gaussian, shown here as a dashed line as guide to the eye. Due to the high nonlinearity of field emission, the amplitude of the current rapidly drops as we move away from the focus. The dotted purple line shows the expected phase shift for a monochromatic beam with the same central wavelength and Rayleigh range as our laser. The agreement of the experimental data with the monochromatic Gouy phase shift is also confirmed by fitting the data with the generalized broadband model (pink dashed line) [17], [18], from which we extract $g_{\phi_{I}}=0.0\pm 0.3$ _._ The shaded pink area shows the broadband phase shift for $\left|g\right|\leq 0.3$ .

At a given position of the nanoantenna, the field-induced current is recorded while varying the CEP over more than 2π. As shown in Figure 2(b), the current undergoes a full oscillation consistent with the CEP sweep. By fitting the data with a sinusoidal function, we can extract the amplitude and phase *ϕ*_*I*_ of the current oscillation, with the related uncertainties. In a previous work, we have shown that, under our experimental conditions, the CEP dependence of field-emission from symmetric single bowtie nanoantennas is independent of the laser intensity [26]. The value of *ϕ*_*I*_ is, therefore, directly related to the CEP at the beginning of the sweep. The Gouy phase shift experienced by the driving electric field transients will be reflected in the measured value of *ϕ*_*I*_. Investigating how the position of the nanoantenna along the focal axis affects the value of *ϕ*_*I*_ allows us to assess the role played by the Gouy phase in the field-emission process.

## 3 Results

**Figure 3:**
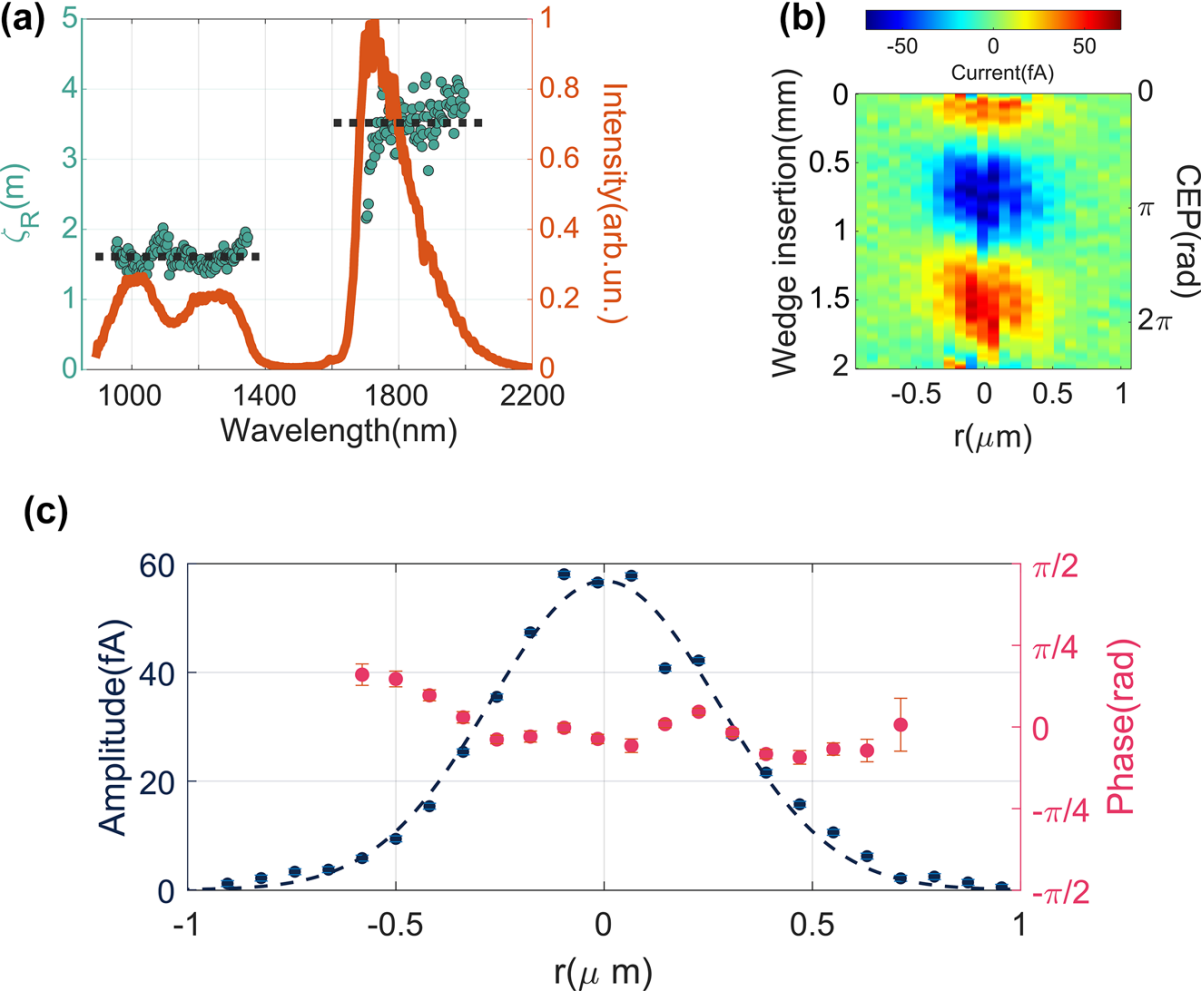
Measurement of the input Rayleigh range ζ_R_ and evolution of *ϕ*_*I*_across the focal plane. (a) Wavelength dependence of *ζ*_*R*_, the Rayleigh range of the input beam prior to focusing. The Rayleigh range is almost independent of wavelength from 1000 nm to 1400 nm and from 1800 nm to 2000 nm (as guide to the eye, we show the average as a black dotted line) the red curve represents the single-cycle laser spectrum. (b, c) Measurement of *ϕ*_*I*_ as the position of the nanoantenna is transversally scanned across the focal plane. (b) 2d map of the field-emitted current measured as a function of wedge insertion and transversal displacement from the focus. (c) Amplitude and phase of the current oscillation, extracted by fitting the experimental data at each position *r* with a sinusoidal function.

By repeating the procedure previously described for different positions of the nanoantenna, we can study the spatial dependence of *ϕ*_*I*_, both along the focal direction and in the transverse plane. The results of the scan along the focal axis are shown in Figure 2. Because of the highly nonlinear nature of field emission, the amplitude of the current rapidly drops as we move away from the focal plane. Despite this, we are successfully able to track the evolution of *ϕ*_*I*_ for roughly 10 μm around the focus, enough to sample a good range of the overall phase shift, given that the Rayleigh range *z*_*R*_ at the central wavelength amounts to 7.4 μm. The value of *z*_*R*_ cannot be measured directly, since our spectrum features a gap around the central wavelength (see Figure 3(a)). We have, therefore, estimated *z*_*R*_ from the expected waist at the central wavelength, assuming Gaussian propagation of the focused beam and taking into account the numerical aperture of the focusing objective. In Figure 2(c), we compare the measured evolution of *ϕ*_*I*_ with the Gouy phase shift expected for a monochromatic wave featuring the same Rayleigh range and central wavelength as our beam. The monochromatic phase shift matches well with our experimental data. To verify this agreement, we also fit the experimental data with the broadband phase shift model, from which we can estimate $g_{\phi_{I}}=0.0\pm 0.3$ . Here, we have used the notation $g_{\phi_{I}}$ to emphasize the fact that we are applying the broadband phase shift model to the measured values of *ϕ*_*I*_. The fact that $g_{\phi_{I}}≃0$ is a further indication that, as we move the nanoantenna along the focus, *ϕ*_*I*_ follows the phase shift of a monochromatic Gaussian beam at the central wavelength of the laser, despite the octave-spanning spectral bandwidth of the laser pulses, the plasmonic reshaping of the electric field at the nano-junction, and the extreme nonlinearity of field emission.

To understand how the Gouy shift affects the field-emission process, we need to compare the evolution of *ϕ*_*I*_ with that of the CEP. To obtain a realistic estimate of the CEP shift of our pulses, we use the broadband phase shift model proposed by Porras [18], which predicts that the CEP evolution is governed by the frequency dispersion of the Rayleigh range of the input beam. To estimate the CEP evolution, we have measured the wavelength-dependent waist size of the input beam. To perform this characterization, we have mounted a razor blade on a translation stage, placed it at the waist position of the input beam and acquired a series of spectra, each corresponding to a different razor-blade insertion into the beam path along the polarization direction. From the waist, we have then estimated the Rayleigh range associated to the different spectral components of our input beam. As shown in Figure 3(a), the Rayleigh range does not show a clear wavelength dependence in the range 1–1.4 μm and 1.8–2.0 μm. Note that the gap in our measurement corresponds to the gap of the laser spectrum. We attribute the jump in Rayleigh range to the fact that, in order to optimize pulse compression, the red and blue portions of our spectrum are spatially separated in a three-prism compressor. Because of the gap at the central wavelength of our spectrum, we cannot apply equation (2) to our data to directly estimate *g*_*CEP*_. However, the physical meaning of *g*_*CEP*_ is to characterize the dispersion of the Rayleigh range. Where the laser spectrum has non-negligible intensity, the Rayleigh range is essentially nondispersive. This is expected and reflects the fact that the octave-spanning pulses originate from a guided mode in an optical fiber. In this sense, we can estimate that *g*_*CEP*_ ≃ 0. In turn, this means that also the CEP of the focused pulses is expected to follow the Gouy shift at the central wavelength. Here, we have called the measured Porras factor *g*_*CEP*_ to stress that this is the quantity that governs the evolution of the CEP, while the previously estimated $g_{\phi_{I}}$ describes the behavior of *ϕ*_*I*_. Interestingly, this measurement suggests a possible way to control the CEP evolution of a broadband laser pulse upon focusing. As shown in [22], by using spatial modulators, it is possible to engineer the waist (and therefore the Rayleigh range) of the different spectral components of the input laser beam, thus matching the value of *g*_*CEP*_ to the specific needs of the experiment.

Surprisingly, the evolution of *ϕ*_*I*_ closely follows the shift of the CEP. This indicates that the field emission at the nano-junction is linearly affected by the CEP shift of the focused free-space laser field, despite the plasmonic reshaping and the extreme nonlinearity of field emission. To explain this behavior, one would need to simultaneously consider many elements, like the exact shape of the field transient at the nano-junction, the precise mechanism of electron tunneling out of the antenna, and the subsequent ballistic motion of the electrons within the gap region. This fact makes a realistic modeling extremely complex and beyond the scope of this work. Experimentally, we can observe that various sources of nonlinearity in the phase dependence of the tunneling current balance each other and result in the overall linear correspondence between the CEP and *ϕ*_*I*_ we have measured.

The linearity between *ϕ*_*I*_ and the CEP suggests that single bowtie nanoantennas can be an interesting platform to develop phase-sensitive detectors. With such devices, one could map the spatial dependence of the CEP and pave the wave toward Gouy phase engineering. In particular, plasmonic CEP-sensitive detectors like gold bowtie nanoantennas can be particularly interesting when dealing with pulse energies below 1 nJ. To our knowledge, this is in fact the first demonstration of CEP spatial characterization of pulses with pJ energy content. To further test single-bowtie nanoantennas as possible phase-sensitive detectors, we investigated the spatial dependence of *ϕ*_*I*_ along the focal plane (see Figure 3(c)). As predicted by the broadband phase shift model for *z* → 0, we could not detect a significative phase shift in the transversal direction. To assess the limitations of a hypothetical device, we have to consider that a nanoantenna acts as an electromagnetic funnel. This does not affect the spatial resolution along the focal axis, since the thickness of the antenna, here on the order of a few tens of nanometers, is much smaller than the Rayleigh range of any typical beam. The longitudinal resolution is, therefore, essentially limited only by the specifications of the stage employed for the scan. Instead, the transversal resolution is limited by the cross section of the nanoantenna, which in this case corresponds to roughly 300 nm. In fact, the part of the laser intensity that illuminates the antenna gets concentrated at the nanoantenna junction. In this picture, the CEP of the locally enhanced electric field can, therefore, be regarded as the average of the free-space CEP over the finite dimension of the nanoantenna. As a result, CEP variations happening on length scales smaller and comparable to the nanoantenna dimensions average out at the junction and cannot be observed in the field-emission process. Despite this, the lateral resolution achievable with our antenna is still sufficient to characterize the phase of a tightly focused laser beam with a spatial resolution comparable to the wavelength, as shown in Figure 3(c).

## 4 Conclusions

In conclusion, we have studied how the spatial dependence of the CEP of single-cycle laser pulses affects the field emission of electrons from a plasmonic nanoantenna. We have tracked the phase *ϕ*_*I*_ of the field-emitted current as the nanoantenna is moved across the focal plane. Surprisingly, the behavior of *ϕ*_*I*_ can be adequately described within the monochromatic Gouy model, despite the octave-spanning bandwidth of the driving laser pulses and the extreme nonlinearity of field emission.

We have compared this result with the expected free-space CEP shift and concluded that *ϕ*_*I*_ linearly follows the CEP of the driving laser field. Interestingly, this result suggests that individual plasmonic bowtie nanoantennas are a good candidate system to develop phase-sensitive detectors for few-cycle laser pulses with pulse energies down to the pJ level. Such detectors can be novel tools that allow to engineer precisely the Gouy phase of optical pulses for experiments where the spatial dependence of the CEP can be harnessed as an additional degree of freedom.

## Supplementary Material

Supplementary Material Details
