# Supplementary material for: Gouy phase effects on photocurrents in plasmonic nanogaps driven by single-cycle pulses
Source: Nanophotonics. 2024 Apr 15;13(15):2803–9. doi: 10.1515/nanoph-2023-0897 (PMC11223509; doi:10.1515/nanoph-2023-0897)
Supplement: Supplementary file 1 — Supplementary Material Details [file j_nanoph-2023-0897_suppl_001.pdf]

Research Article

Andrea Rossetti, Matthias Falk, Alfred Leitenstorfer, Daniele Brida and Markus Ludwig

Gouy Phase Effects on Photocurrents in Plasmonic Nanogaps driven by Single-Cycle Pulses

Supplementary Material:

We use the knife-edge method to characterize the movement of the nanopositioner with respect to the optical axis, both in the z (longitudinal) and x (transversal) direction. This is achieved by scanning the beam across a lithographically defined sharp metallic edge in the vicinity of the bowtie antenna and acquiring the transmitted intensity curve. To identify the focal plane, we repeat the knife-edge measurement for different positions of the sample along the focal axis. The results of this measurement are shown in the left panel of figure S1. We fit the transmitted intensity curves with an error function and obtain the spot size as a function of the focal axis position. We then fit the values of spot size assuming Gaussian beam propagation around the focal plane (see left panel figure S1). In particular we fit the spot size with the function:

$$w(z) = w_0\sqrt{1+\left(\frac{z}{z_R}\right)^2}$$

where w_0 is the waist, z_R the Rayleigh range and z_0 the focal position. From this fit we can locate the focal plane with an associated error of 1.6µm for a 95% confidence interval. This should be considered an upper limit on the error we make in identifying the focal plane. As mentioned above, in the actual experiment we locate the focal plane by optimizing the amplitude of the current oscillation. Since the current depends nonlinearly on the laser intensity, the precision we expect to achieve using this last method should overtake the one achieved using the knife-edge method. Therefore, we believe that the actual uncertainty that we have on the focal plane position is less than 1.6µm.

To verify that the transversal movement of fig. 3c indeed lies in the focal plane, we have performed a series of knife-edge measurements, where we translate the sharp metallic edge along the x direction, with z fixed at the focal plane. If the x-axis of the positioner does not lie in the focal plane, we expect to observe a change in spot size for different x positions. The results of this measurement are shown in the right panel of figure S1. We obtain that the spot size changes by roughly 200nm over a 20µm scan. This means that we observe only a 1% change in spot size for the <2µm transversal displacement considered in Fig. 3c. We conclude that the movement of the sample in the transversal is decoupled from that along the focal axis.

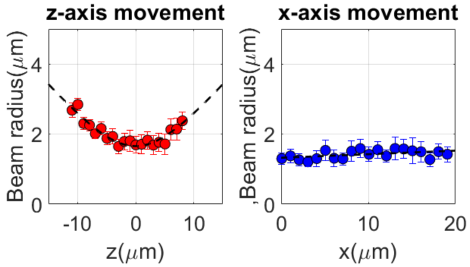

Figure S1: (left) Identification of the focal plane using the knife-edge method. (right) Beam radius measured for different stage-positions along the x -axis. The fact that the spot size is practically constant over a 20µm confirms that the movement done in fig. 3c lies in the focal plane.

Corresponding authors: daniele.brida@uni.lu; markus.ludwig@desy.de

Andrea Rossetti: Department of Physics and Materials Science, University of Luxembourg, Luxembourg, Luxembourg
Matthias Falk: Department of Physics and Center for Applied Photonics, University of Konstanz, Konstanz, Germany
Alfred Leitenstorfer: Department of Physics and Center for Applied Photonics, University of Konstanz, Konstanz, Germany
Daniele Brida: Department of Physics and Materials Science, University of Luxembourg, Luxembourg, Luxembourg
Markus Ludwig, Deutsches Elektronen-Synchrotron (DESY), Hamburg, Germany
